# Functional Characterisation of NF-YCs in True Leaf Biomass Accumulation

**DOI:** 10.3390/plants15121789

**Published:** 2026-06-10

**Authors:** Shuhan Yu, Xumin Wang, Bowei Zhu, Yujiao Song, Guodong Zhao, Yaping Song, Tongsheng Zhao, Gang Niu, Yingjie Wang, Dong Li, Da Zhang

**Affiliations:** 1 Yantai Key Laboratory of Molecular Breeding for High-Yield and Stress-Resistant Crops and Efficient Cultivation, The Engineering Research Institute of Agriculture and Forestry, Ludong University, Yantai 264025, China; 13515357072@163.com (S.Y.); 15939492734@163.com (Y.S.); 2 Yantai Technology Center of Characteristic Plant Gene Editing & Germplasm Innovation, College of Horticulture, Ludong University, Yantai 264025, China; 3 Changli Institute of Pomology, Hebei Academy of Agriculture and Forestry Sciences, Qinhuangdao 066600, China; minr@nwafu.edu.cn (X.W.); 18331561549@163.com (B.Z.); guodong19823@163.com (G.Z.); syp0526@126.com (Y.S.); tshzh71@163.com (T.Z.); niug@nwafu.edu.cn (G.N.); 17733503931@163.com (Y.W.); 4 State Key Laboratory for Crop Stress Resistance and High-Efficiency Production, Northwest A&F University, Yangling 712100, China

**Keywords:** NF-YC, leaf biomass, HY5, *A. thaliana*

## Abstract

Leaf biomass accumulation is a critical determinant of photosynthetic capacity and crop productivity. In *Arabidopsis thaliana*, multiple hormonal and environmental pathways, including brassinosteroid (BR), auxin and light signaling, as well as functional proteins such as TNY (TINY), SAUR21 (SMALL AUXIN UP-RNA21) and HY5 (ELONGATED HYPOCOTYL 5), play important roles in regulating leaf growth. However, the precise regulatory mechanisms integrating these factors during leaf biomass accumulation remain incompletely understood. Herein, we showed that *NF-YC3*/*4*/*9*, members of the NUCLEAR FACTOR Y subunit C family, were required for normal leaf cell expansion. Loss-of-function mutation of *NF-YC3*/*4*/*9* (*nf-ycT*) resulted in significantly smaller true leaves with reduced leaf cell expansion. NF-YC9 directly regulated the expression of *HY5*, *HYH*, and *SAUR21* and indirectly regulated the expression of *TNY*. These results help reveal the function of *NF-YCs* in leaf growth and provide insights into the regulation of hormonal and transcriptional networks controlling leaf biomass accumulation in *A. thaliana*.

## 1. Introduction

Leaves serve as the main photosynthetic structures in plants, efficiently transforming solar energy into biological energy to support plant growth and development [1]. Accumulation of leaf biomass, encompassing the increased size and number of true leaves, before flowering boosts photosynthetic performance and stimulates seed development, consequently improving seed production and quality [2]. Moreover, the young leaves and bolting stalks of *Brassica* crops are also important sources of diverse nutrients and phytochemicals beneficial to human health [3]. However, the precise regulatory mechanisms integrating multiple hormonal and transcriptional pathways during leaf biomass accumulation remain incompletely understood.

The close evolutionary relationship between *A. thaliana* and *Brassica* crops makes it an ideal model system for studying leaf biomass accumulation [4,5]. Leaf initiation occurs at the peripheral regions of the shoot apical meristem. A cylindrical primordium flattens to form the leaf blade. Following the cessation of cell proliferation, cells begin to enlarge and expansion predominates as the principal driver of organ growth [6]. In the regulatory network controlling leaf biomass accumulation in *A. thaliana*, factors that mediate hormonal and environmental signals play critical roles [2,7,8,9]. Of these, TNY was demonstrated to negatively regulate leaf growth, likely by interfering with brassinosteroid (BR)-mediated cell elongation processes [10]. At the post-translational level, BIN2 (BRASSINOSTEROID-INSENSITIVE 2) phosphorylates and stabilizes TNY [10]. Besides BRs, auxin is also recognized to promote cell elongation through enhanced wall extensibility [11]. Auxin receptor ABP1 (AUXIN BINDING PROTEIN 1) promotes cell expansion by regulating cell wall remodeling gene expression and modulating xyloglucan structure. [12]. Among auxin-induced genes, *SAURs* form the largest family, with 79 members in *A. thaliana* and comparable numbers in other plants [13]. The expression of numerous auxin-inducible *SAURs* is tightly linked to ongoing cell expansion [14]. Members of the *SAUR19* subfamily act as positive regulators of cell expansion. Their overexpression promotes cell enlargement, whereas their knockdown reduces growth. These effects are likely mediated by modulation of auxin transport [15], and constitutive *SAUR19* expression in tomato promotes auxin-independent elongation growth [14]. bZIP transcription factor (TF) HY5 plays a central role in the light signaling pathway, promoting photomorphogenesis through direct or indirect interactions with multiple key factors, including B-box-containing proteins, and regulating nearly one-third of the genes in the Arabidopsis genome [16,17,18,19]. Moreover, besides having shorter hypocotyls, *HY5* overexpression lines also showed markedly smaller leaves, which are associated with its interaction with ABI5 (ABA INSENSITIVE 5) and alterations in ABA signaling [20]. HYH (HY5-HOMOLOG) has been shown to share overlapping functions with HY5 and act redundantly in regulating plant growth and development [21]. Nevertheless, the precise regulatory mechanisms by which these factors orchestrate leaf biomass accumulation remain to be fully elucidated.

In higher plants, the NF-YCs (NUCLEAR FACTORY C proteins) serve as crucial regulators involved in diverse developmental processes and stress response. In *A. thaliana*, NF-YCs are required for CONSTANS-mediated, photoperiod-dependent flowering regulation [22]. The NF-YC–RGL2 (RGA-LIKE 2) module mediates crosstalk between GA and ABA signaling to modulate seed germination [23]. NF-YCs and ARP6 (ACTIN-RELATED PROTEIN 6) cooperatively suppress hypocotyl elongation by mediating H2A.Z deposition at target IAAs during photomorphogenesis [24]. NF-YC9 overexpression increases seedling sensitivity to abscisic acid [25]. The QQS orphan gene and its interactor NF-YC4 confer enhanced resistance to pests and pathogens [26]. Jasmonate signaling enhances salt stress tolerance via the NF-YC9-YA1-YB2 trimeric complex [27]. SUMOylation promotes the assembly of the NF-YC10-YB3 complex to enhance thermotolerance [28]. NF-YCs cooperate with ABF3 (ABSCISIC ACID RESPONSIVE ELEMENTS-BINDING FACTOR 3) and ABF4 to modulate *SOC1* (*SUPPRESSOR OF OVEREXPRESSION OF CO 1*) expression, thereby mediating drought-accelerated flowering [29]. NF-YCs also negatively regulate seed germination in response to salinity stress [30]. However, the role of NF-YCs in regulating leaf biomass accumulation remains poorly characterized, and their functional relationship with the aforementioned factors remains to be elucidated.

In this study, it was found that nf-*ycT* (*nf-yc3-2 nf-yc4-1 nf-yc9-1*) mutant true leaves were significantly smaller than those of the wild type, with reduced leaf cell expansion. *nf-ycT* mutation affected the expression of a series of genes involved in hormone response and photomorphogenesis, including transcription factors *HY5*, *HYH*, and *TNY* and hormone-related *BAT1* (*BR-RELATED ACYLTRANSFERASE 1*), *GRACE* (*GERMINATION REPRESSION AND CELL EXPANSION RECEPTOR-LIKE KINASE*), and *SAUR21*. Notably, NF-YC9 directly regulates the expression of *HY5*, *HYH*, and *SAUR21*. These results indicate that NF-YCs function as important modulators linking hormone regulation and leaf biomass accumulation.

## 2. Materials and Methods

### 2.1. Plant Materials and Growth Conditions

The *nf-ycT* triple mutant (*nf-yc3-2 nf-yc4-1 nf-yc9-1*) in the Col-0 background and complementation line in *nf-ycT* background (*pNF-YC9:NF-YC9-FLAG;nf-ycT*) have been utilized previously [31]. Seeds were stratified at 4 °C for 2 days prior to transfer to a growth chamber. Detailed growth conditions for all plants were as previously reported [30]. Briefly, one-week-old seedlings cultivated on 1/2 MS medium were transferred to 0.6 L pots containing a 4:1 (*v*/*v*) mixture of nutrient soil and autoclaved vermiculite, and then maintained under 16 h light (natural daylight supplemented with LED lamps, average intensity 160 μmol·m^−2^·s^−1^)/8 h dark cycles with 60 ± 5% relative humidity at 22 °C.

### 2.2. Morphological Observation

Randomly selected whole plants and true leaves were photographed with a Canon camera (Canon EOS 7D, Tokyo, Japan). To examine subepidermal cells, the middle region of true leaves was peeled to expose the subepidermal layer, and images were captured using an Olympus BX63 microscope (Olympus BX63, Tokyo, Japan). Leaf area and cell area were quantified using ImageJ software (version 1.54; National Institutes of Health, Bethesda, MD, USA). The cell number per leaf was estimated by dividing the average leaf area by the average cell area.

### 2.3. RNA-Seq Experiment and Data Analysis

RNA-seq analysis was performed on 10-day-old seedlings cultivated on 1/2 MS medium of the *nf-ycT* and Col-0. For each genotype, three biological replicates were submitted to BGI-Tech (Shenzhen, China) for sequencing according to their standard pipeline (http://bgitechsolutions.com/sequencing/45, accessed on 14 March 2025). In brief, paired-end reads were generated on a BGISEQ-500 platform. Low-quality reads were discarded using SOAPnuke (v1.4.0). The reference genome of *A. thaliana* was retrieved from TAIR (https://www.arabidopsis.org/, accessed on 14 March 2025), and read alignment was performed with HISAT (http://www.ccb.jhu.edu/software/hisat, accessed on 14 March 2025). Differentially expressed genes (DEGs) were defined by |log_2_ fold change| ≥ 1 and false discovery rate (FDR) ≤ 0.05. Functional enrichment analysis of gene ontologies (GO) was carried out via the DAVID platform (https://davidbioinformatics.nih.gov/, accessed on 25 November 2025).

### 2.4. Gene Expression Analysis

For each genotype, total RNA was extracted using the Plant RNA Kit (Transgen Biotech Co. Ltd., Beijing, China) and subsequently converted to cDNA with EasyScript One-Step gDNA Removal and cDNA Synthesis SuperMix (Transgen Biotech Co. Ltd., Beijing, China). RT-qPCR was performed on an Applied Biosystems StepOne Plus™ instrument (Applied Biosystems, Waltham, MA, USA) using SYBR Green Master Mix (Transgen Biotech Co. Ltd., Beijing, China) with three biological replicates. Transcript abundance was calculated relative to the internal reference gene *EF1αA4*. Primer sequences are provided in Supplementary Table S1.

### 2.5. ChIP-qPCR Assay

ChIP-qPCR was performed following a previously described protocol [30]. Briefly, seedlings of *pNF-YC9:NF-YC9-FLAG;nf-ycT* and Col-0 were vacuum-infiltrated with 1% formaldehyde on ice for 15 min to cross-link proteins, and the reaction was quenched by adding 125 mM glycine for another 5 min. After two washes with distilled water, the samples were snap-frozen in liquid nitrogen. Isolated chromatin was sonicated to produce DNA fragments ranging from 250 to 700 bp. The sheared chromatin was immunoprecipitated overnight at 4 °C using Pierce anti-FLAG magnetic beads (Thermo Fisher, Waltham, MA, USA). The beads were collected on a magnetic stand, washed, and the immune complexes were eluted twice. The eluates were then reverse-crosslinked in 5 M NaCl at 65 °C for 10 h, followed by protein digestion with 1 M Tris-HCl (pH 6.5), 0.5 M EDTA, and 1.5 μL proteinase K (20 mg/mL) at 45 °C for 1 h. DNA was purified via phenol/chloroform/isoamyl alcohol (25:24:1) extraction and stored at −80 °C. Relative enrichment of each fragment was determined via RT-qPCR. Three technical replicates were run per independent experiment, and the entire experiment was repeated three times. Enrichment values were normalized to the internal control *EF1αA4* (ratio of *pNF-YC9:NF-YC9-FLAG;nf-ycT* to Col-0), with TUB2 serving as a negative control. All ChIP-qPCR primers are listed in Supplementary Table S1.

### 2.6. Dual-Luciferase Assay

Dual-luciferase reporter assays were carried out as previously described [30]. Effector constructs carrying GFP or NF-YC9, along with reporter constructs containing the promoters of *HY5*, *HYH*, or *SAUR21*, were introduced into *Agrobacterium tumefaciens* strain GV3101 harboring pSoup-P19 (Weidi Biotechnology, Shanghai, China). Plasmid mixtures were infiltrated into young leaves of 4-week-old *Nicotiana benthamiana* plants. Infiltrated plants were then placed in a growth chamber under long-day conditions (16 h light/8 h dark) at 22 °C for three days. Total protein was extracted from the infiltrated leaf tissues using Cell Lysis Buffer (Yeasen Biotechnology, Shanghai, China). Firefly luciferase (LUC) and *Renilla* luciferase (REN, internal control) activities were measured with a Luciferase Reporter Gene Assay Kit (Yeasen Biotechnology, Shanghai, China) on a multifunctional microplate reader (Tecan, Männedorf, Switzerland). Nine independent biological replicates were examined for each combination. Primers used for vector construction are listed in Supplementary Table S1.

### 2.7. Statistical Analyses

A completely randomized design was adopted. Data are presented as mean ± SD. Statistical comparisons between two groups were performed using a two-tailed Student’s *t*-test. A significance level of *p* < 0.05 was considered statistically significant.

## 3. Results

### 3.1. nf-ycT Triple Mutant Exhibits Reduced True Leaf Size

Previous β-glucuronidase (GUS) reporter assays in *A*. *thaliana* seedlings revealed that *NF-YC3*, *NF-YC4*, and *NF-YC9* exhibited strong and consistent leaf mesophyll and vascular expression [22]. Therefore, NF-YCs were selected as potential targets in this study to investigate their roles in the leaf growth process. Considering the potential functional redundancy among NF-YCs, the *nf-ycT* triple mutant (*nf-yc3-2 nf-yc4-1 nf-yc9-1*) in the Col-0 background was selected for phenotypic analysis.

**Figure 1:**
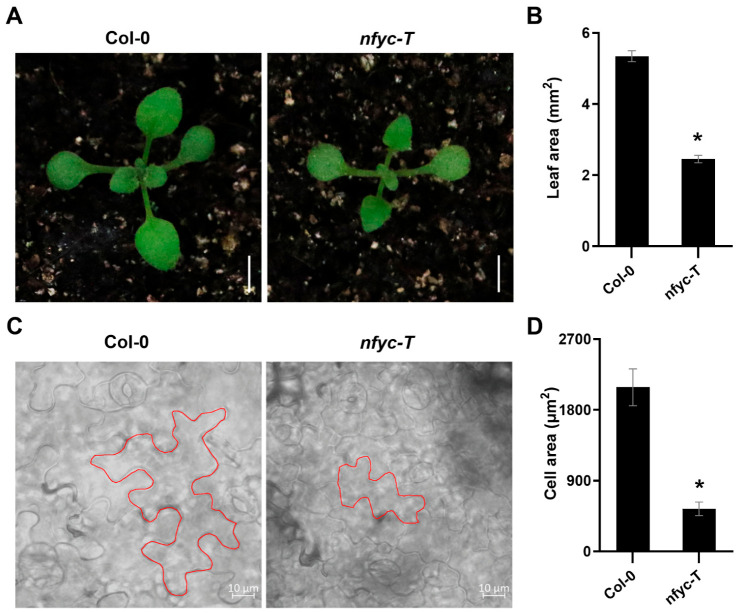
NF-YCs function as positive regulators of true leaf biomass accumulation. (**A**) Morphological observation of whole plants and developing true leaves of *nf-ycT* and Col-0 at 14 DAG. Scale bars = 2 mm (**B**) Statistical analysis of the first true leaf area in *nf-ycT* versus Col-0 at 14 DAG. Data are presented as means ± SD (*n* = 7). Asterisks mark significant differences between *nf-ycT* and Col-0 (two-tailed paired Student’s *t*-test; *p* ≤ 0.05). (**C**) Microscopic observation of subepidermal cells in the middle region of the first true leaves of *nf-ycT* and Col-0 at 14 DAG. Representative intact cells are outlined by red lines. Scale bars = 10 μm. (**D**) Comparison of mean cell area in the first true leaves of *nf-ycT* and Col-0 at 14 DAG. Data are presented as means ± SD (*n* = 7). Asterisks denote significant differences compared with Col-0 (two-tailed paired Student’s *t*-test; *p* ≤ 0.05).

The *nf-ycT* were grown in a growth chamber together with the wild-type control. Phenotypic observation revealed that at about 14 days after germination (DAG), *nf-ycT* seedlings were significantly smaller than Col-0 (Figure 1A). Leaf area measurement further showed that the true leaf area of *nf-ycT* was approximately half that of Col-0 (Figure 1B). Because cell size is a key determinant of leaf size, the subepidermal cell area was measured in *nf-ycT* and Col-0 leaves. The cell area was significantly reduced in *nf-ycT* leaves (Figure 1C,D). Moreover, *nf-ycT* showed significantly reduced true leaf biomass relative to Col-0, as measured by total true leaf fresh weight at 14 DAG (Supplementary Figure S1), despite a slight increase in leaf cell number (Supplementary Figure S2). Collectively, these results suggest that *NF-YCs* promote true leaf biomass accumulation by increasing cell area during leaf development in *A. thaliana*.

### 3.2. Transcriptome Profiling of the nf-ycT Quadruple Mutant

**Figure 2:**
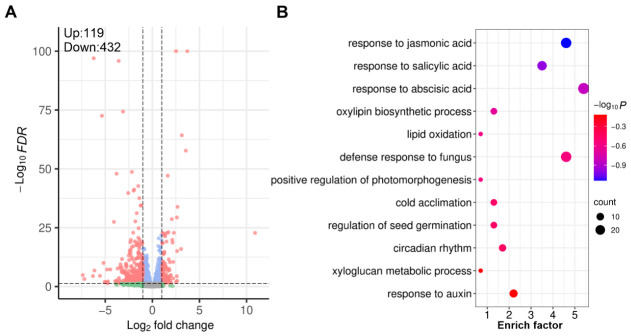
Transcriptomic profiling reveals leaf growth-associated genes affected by *nf-ycT* mutation. (**A**) Volcano plots showing global gene expression patterns in *nf-ycT* true leaves relative to Col-0 at 10 DAG. Red dots indicate differentially expressed genes (DEGs) meeting the criteria of |log_2_ fold change (FC)| ≥ 1 and false discovery rate (FDR) ≤ 0.05. (**B**) GO enrichment analysis performed on DEGs from *nf-ycT* true leaves compared with Col-0.

To understand the role of *NF-YCs* in promoting true leaf biomass accumulation, a genome-wide transcriptome analysis was conducted using true leaves of *nf-ycT* and Col-0 harvested at 10 DAG. The transcriptional changes identified in this assay could promote, at least in part, a deeper insight into the regulatory circuits underlying the *NF-YC*-controlled regulation of leaf development. The assay identified 551 differentially expressed genes (DEGs), among which 432 were downregulated and 119 were upregulated in *nf-ycT* leaves (Figure 2A; Supplementary Table S2).

To gain insight into the functional categories and pathways perturbed by the loss of NF-YC function, GO enrichment analysis was conducted on the identified DEGs. GO enrichment analysis revealed that the DEGs were significantly enriched in functional categories associated with responses to multiple phytohormones (including jasmonic acid (JA), salicylic acid (SA), abscisic acid (ABA), and auxin), abiotic and biotic stresses (cold and fungal infection), as well as photomorphogenesis, seed germination, and xyloglucan metabolism (Figure 2B). Collectively, these findings imply that *NF-YCs* regulate leaf growth by potentially integrating multiple regulatory networks, including those associated with phytohormone signaling, light-responsive pathways, and cell wall metabolism.

### 3.3. Verification of DEGs by qRT-PCR

**Figure 3:**
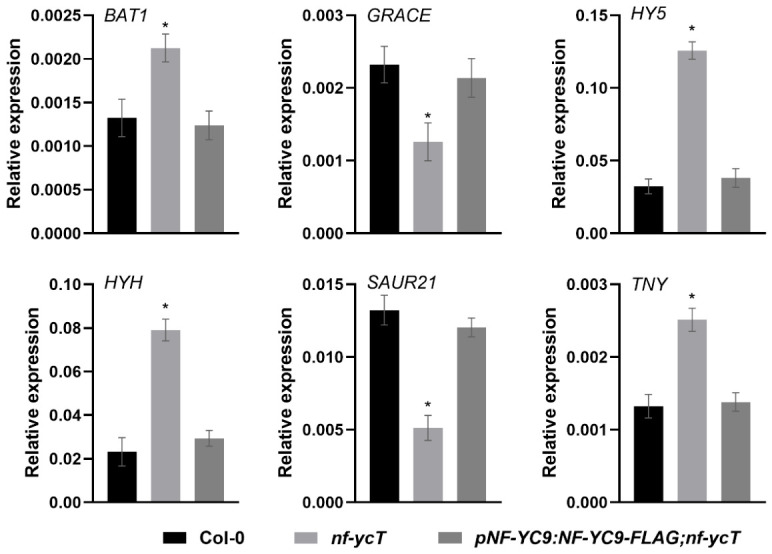
RT-qPCR quantification of transcript levels of leaf growth-associated DEGs in the *nf-ycT*, *pNF-YC9:NF-YC9-FLAG;nf-ycT* and Col-0 plants. Data were normalized to *EF1αA4* as an internal reference. Values are presented as means ± SD (*n* = 3). Asterisks denote significant differences relative to Col-0 (two-tailed paired Student’s *t* test, *p* ≤ 0.05).

Based on the above GO enrichment results, some hub genes associated with the key biological processes were selected and analyzed. The expression of JA-signaling-related genes *JAZ3* (*JASMONATE-ZIM-DOMAIN PROTEIN 3*), *JAZ8*, *JAZ7*, and *JAZ10* was downregulated, whereas the ethylene-related gene *TNY2* was upregulated. Among GA-signaling components, *RGL2* was upregulated. Auxin-related genes *SAUR21*, *IAA1* (*INDOLEACETIC ACID-INDUCED PROTEIN 1*), and *IAA5* were downregulated, whereas *PIN5* (*PIN-FORMED 5*) was upregulated. For BR signaling, both *BAT1* and *TNY* were upregulated. ABA-associated genes *ABI5* and *CYP707A3* were also upregulated. Stress-responsive genes *HSFA2* (*HEAT SHOCK TRANSCRIPTION FACTOR A2*), *CBF1* (*C-REPEAT/DRE BINDING FACTOR 1*), and *CBF2* were upregulated, while *P5CS1* was downregulated. Xyloglucan metabolic genes *XTH24* (*xyloglucan endotransglucosylase/hydrolase 24*) and *XTH25* were downregulated. Light-responsive genes *HY5* and *HYH* were upregulated, whereas the fungus-responsive gene *OPR3* (*OXOPHYTODIENOATE-REDUCTASE 3*) was downregulated (Supplementary Table S2). Furthermore, based on their functional characterization in previously published studies, candidate genes involved in growth regulation, including *BAT1*, *GRACE*, *HY5*, *HYH*, *SAUR21*, and *TNY*, were selected for qRT-PCR validation of their expression patterns in true leaves of *nf-ycT*, *pNF-YC9:NF-YC9-FLAG;nf-ycT* and Col-0 plants at 10 DAG (Figure 3). As expected, the qRT-PCR results for these six genes in *nf-ycT* and Col-0 were consistent with the transcriptome data (Supplementary Table S2), and their expression levels could be restored to the wild-type level in the *pNF-YC9:NF-YC9-FLAG;nf-ycT* line. The NF-YC9-FLAG fusion protein retained the same biological function as NF-YC9 in true leaves (Supplementary Figure S3). Collectively, the transcriptome approach proves to be a powerful means to uncover genes downstream of NF-YCs.

### 3.4. NF-YC9 Directly Binds to the Promoters of HY5, HYH, and SAUR21

**Figure 4:**
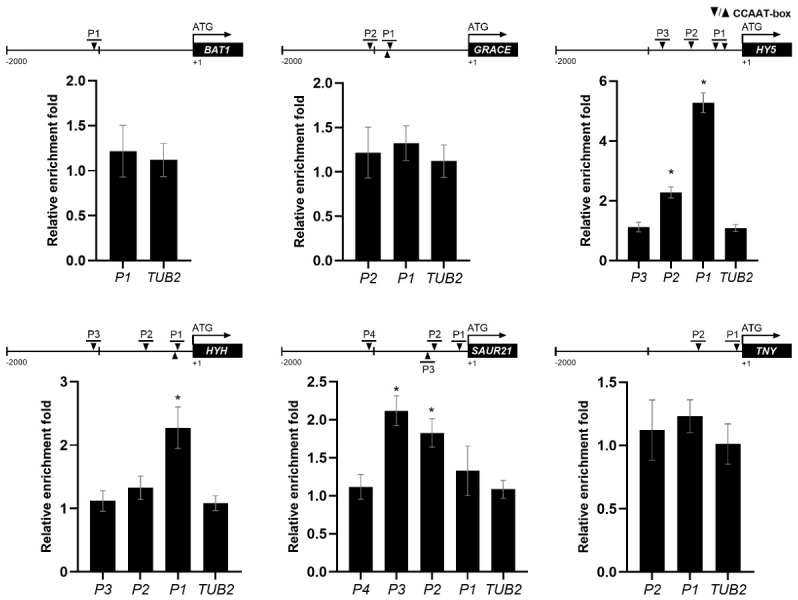
NF-YC9 targets the *HY5*, *HYH*, and *SAUR21* promoters. ChIP-qPCR analysis of NF-YC9 binding to the promoter regions of *BAT1*, *GRACE*, *HY5*, *HYH*, *SAUR21*, and *TNY* in true leaves. The fold enrichment of each fragment was calculated by first normalizing the target DNA amount to a genomic fragment of *EF1αA4* (internal control) and then normalizing the value obtained for *pNF-YC9:NF-YC9-FLAG;nf-ycT* to that of Col-0. The *TUB2* fragment served as a negative control. Values are means ± SD (*n* = 3). Asterisks indicate significant differences relative to the enrichment of the *TUB2* fragment (two-tailed paired Student’s *t*-test, *p* ≤ 0.05). The schematic representation above the bars illustrates the promoters with putative CCAAT-box motifs upstream of the ATG start codons. Exons are shown as black boxes, whereas promoter regions are shown as black lines. CCAAT-box motifs are indicated by black triangles. PCR-amplified fragments are indicated by black lines positioned above the CCAAT-box motifs.

NF-YCs are subunits of the trimeric NF-Y complex, which binds selectively to the CCAAT-box motif in eukaryotes. Sequence analysis revealed that the promoters of all six DEGs contained CCAAT-box motifs (Figure 4). To investigate how NF-YCs regulate the expression of these six genes, ChIP-qPCR assays were performed using true leaves of *pNF-YC9:NF-YC9-FLAG;nf-ycT* plants at 14 DAG. The ChIP-qPCR results showed that NF-YC9 was associated with the promoter regions near fragments P1 and P2 of *HY5*, fragments P1 of *HYH*, and fragments P2 and P3 of *SAUR21*, whereas it did not bind to the promoters of *BAT1*, *GRACE*, or *TNY* (Figure 4). These results suggest that *HY5*, *HYH*, and *SAUR21* are direct targets of NF-YC9, but *BAT1*, *GRACE*, and *TNY* are not.

### 3.5. NF-YC9 Directly Regulates the Expression of HY5, HYH, and SAUR21

**Figure 5:**
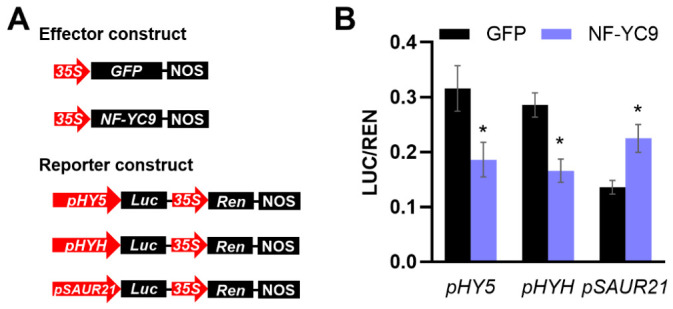
NF-YC9 regulates the transcriptional activities of *HY5*, *HYH*, and *SAUR21*. (**A**) Schematic diagrams depicting the effectors (GFP and NF-YC9) and the reporters carrying the *HY5*, *HYH*, and *SAUR21* promoters. (**B**) Transient dual-luciferase reporter assay. Each reporter construct was co-introduced with the GFP or NF-YC9 effector construct and transiently expressed in leaf cells of 4-week-old *N. benthamiana* plants. The infiltrated plants were maintained in a growth chamber under long-day conditions (16 h light/8 h dark) at 22 °C for 3 days. Renilla luciferase (REN) activity served as an internal control, and the relative LUC activity (LUC/REN) reflected the relative transcriptional activity of the *HY5*, *HYH*, and *SAUR21* promoters. Relative activities were calculated by normalizing to the GFP control. Values are means ± SD (*n* = 9). Asterisks denote significant differences in relative LUC activity between the NF-YC9 effector and the GFP control (two-tailed paired Student’s *t*-test, *p* ≤ 0.05).

Additionally, this work assessed the transcriptional regulation of NF-YC9 on *HY5*, *HYH*, and *SAUR21* via dual-luciferase reporter assay in *N. benthamiana* leaves. Constructs harboring *HY5*, *HYH*, and *SAUR21* promoter-driven LUC, and 35S promoter-driven REN were used as reporters. The pGreenII 62-SK recombinant vectors carrying the CDSs of *GFP* and *NF-YC9* were used as effectors (Figure 5A). The NF-YC9 effector led to a decrease in *ProHY5:LUC* and *ProHYH:LUC* expression and an elevation of *ProSAUR21:LUC* expression relative to the GFP control (Figure 5B). In summary, these results collectively demonstrate that NF-YC9 binds to the promoters of *HY5*, *HYH*, and *SAUR21*, thus directly regulating their expression to promote true leaf biomass accumulation.

## 4. Discussion

Leaves serve as the primary photosynthetic organs in plants. Leaf biomass, determined by leaf number and size, directly impacts photosynthetic efficiency and consequently crop yield and quality [2,32]. As a fellow member of the Brassicaceae family, *A. thaliana* research can shed light on *Brassica* crops important to human consumption [33,34]. However, the molecular basis of leaf biomass accumulation in *A. thaliana* remains elusive.

It is well established that leaf size is governed by cell number and size, and that the pre-flowering increase in true leaf number and size enhances leaf biomass accumulation by promoting photosynthetic efficiency [32,35,36]. The reverse genetic assays in this study showed that the true leaves of *nf-ycT* were significantly smaller than those of Col-0, which might be caused by reduced cell area at 14 DAG (Figure 1). Consistently, the fresh weight of *nf-ycT* true leaves was also lower than that of Col-0 (Supplementary Figure S1). Therefore, *NF-YCs* function as positive regulators of true leaf biomass accumulation. The discrepancy in leaf size phenotypes between our study and that of Kumimoto et al. (2010) may be attributed to differences in developmental stages [22]. Moreover, the strong impact of *nf-ycT* on flowering time subsequently influences late leaf development [22]. Future studies using gene editing to uncouple the flowering defect from the *nf-ycT* mutation would allow a more comprehensive assessment of leaf phenotypes at the mature stage. Notably, the phenotypic difference was only observed in true leaves but not in cotyledons (Figure 1), further indicating that NF-YCs act as key regulators of true leaf development rather than causing a general growth delay. Similarly, recent studies have revealed that TOP1αs, which are also well-known regulators of flowering time, are involved in true leaf biomass accumulation by modulating cell division, without affecting cotyledons [2]. Given their shared roles in the regulation of both flowering time and leaf growth [37,38], whether any functional connection exists between NF-YCs and TOP1αs, such as protein–protein interaction or transcriptional regulation, represents an intriguing avenue for future investigation.

Leaf biomass accumulation is tightly governed by a complex transcriptional regulatory network [39]. Transcriptomic analysis comparing *nf-ycT* and Col-0 revealed that the differentially expressed genes (DEGs) were significantly enriched in multiple leaf growth-related biological processes, such as light and hormone responses (Figure 2). Previous studies reported that NF-YCs, together with RGL2, repress GA-mediated seed germination by promoting *ABI5* expression [23]. In our study, both *RGL2* and *ABI5* were upregulated in the *nf-ycT* mutant (Supplementary Table S2). Given that NF-Ys are important regulators mediating plant development and environmental responses, the opposite role of NF-YCs in ABA signaling between germination and leaf growth may result from spatiotemporal regulation of NF-Y complexes, which consist of various NF-YA/B/C subunits at different developmental stages [40]. Moreover, it has been reported that HY5 shares a substantial set of target genes with NF-YCs [41], and this conclusion is further corroborated by the identification of DEG *ABI5* in the present study (Supplementary Table S2). Taken together with previous findings, this suggests that both NF-YCs and HY5 may act upstream of *ABI5* [20]. Notably, a recent study demonstrated a physical interaction between NF-YC9 and HY5 [42], seemingly further confirming their co-function at the same regulatory tier. However, our study uncovers a novel transcriptional regulatory mode in which NF-YC9 and HY5 constitute a complex feed-forward loop that controls leaf growth (Figure 4). Based on these findings, we propose that NF-YCs may regulate true leaf growth through HY5-mediated light signaling—a possibility that warrants further investigation, for example, by manipulating light quality or photoperiod conditions.

Phytohormones form a complex regulatory network that controls leaf biomass accumulation. Auxin promotes apoplast acidification by activating cell wall-related gene expression and stimulating proton pump synthesis. It also activates PM H^+^-ATPases via phosphorylation of their penultimate threonine residue, further contributing to apoplast acidification [11,43]. Moreover, activation of PM H^+^-ATPase leads to PM hyperpolarization, consequently facilitating solute and water uptake and providing the increased intracellular turgor required for cell expansion [14]. Cell expansion is defined as an increase in cell size and thus plays a fundamental part in plant growth and development [12]. Recent evidence suggests that SAURs can also modulate H^+^ pumps [13]. Therefore, the reduced cell size observed in the *nf-ycT* mutant may be associated with decreased auxin-mediated intracellular turgor. GRACE acts as a positive regulator of cell expansion and organ growth, likely through perceiving a yet-unknown ligand via its structurally distinct island domain [44]. *BAT1* encodes an acyltransferase that regulates endogenous BR levels by acylating brassinolide, castasterone, and typhasterol, and overexpression of *BAT1* leads to typical dwarf growth defects resulting from BR deficiency [45]. On the other hand, JAZs and IAAs function as key repressors of JA and auxin signaling, and their roles in leaf growth regulation, as well as their relationships with NF-YCs, warrant further investigation [46]. The complex patterns of up- and down-regulated genes associated with diverse hormones in the downstream network suggest that additional modulators may be involved in the NF-YC-mediated pathway [23]. Future yeast two-hybrid library screening would therefore be a valuable approach to dissect the interplay between hormone regulation and transcriptional control. In summary, these findings provide insights into the understanding of how NF-YCs coordinate hormonal and light signaling to regulate leaf growth and lay a foundation for breeding high-biomass cultivars to improve crop productivity through biotechnological strategies.

## Data Availability

The raw data supporting the conclusions of this article will be made available by the authors upon request. Sequence data mentioned in this work can be retrieved from the Arabidopsis Genome Initiative database under the following accession numbers: BAT1 (AT4G31910), GRACE (AT1G74360), HY5 (AT5G11260), HYH (AT3G17609), NF-YC3 (AT1G54830), NF-YC4 (AT5G63470), NF-YC9 (AT1G08970), SAUR21 (AT5G18030), TNY (AT5G25810).

## Supplementary Materials

The following supporting information can be downloaded at https://www.mdpi.com/article/10.3390/plants15121789/s1. Supplementary Figure S1. Comparison of total true leaf fresh weight between *nf-ycT* and Col-0. Supplementary Figure S2. Comparison of cell number of first true leaf between *nf-ycT* and Col-0. Supplementary Figure S3. Comparison of leaf area and cell area between Col-0 and *pNF-YC9:NF-YC9-FLAG;nf-ycT* line. Supplementary Table S1. Primers used in this study. Supplementary Table S2. List of differentially expressed genes between nf-ycT and Col-0.
