# Supplementary material for: Functional Characterisation of NF-YCs in True Leaf Biomass Accumulation
Source: Plants (Basel). 2026 Jun 10;15(12):1789. doi: 10.3390/plants15121789 (PMC13306752; doi:10.3390/plants15121789)
Supplement: Supplementary file 1 [file plants-15-01789-s001.zip › plants-4337609-Supplementary Figures.pdf]

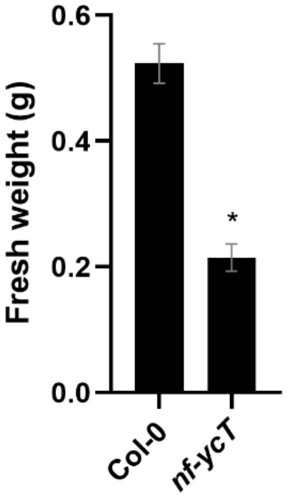

Supplementary Figure S1. Comparison of total true leaf fresh weight between *nf-ycT* and Col-0 (two-tailed paired Student's *t*-test; $P \leqslant 0.05$). Data are presented as means ± SD (n = 7).

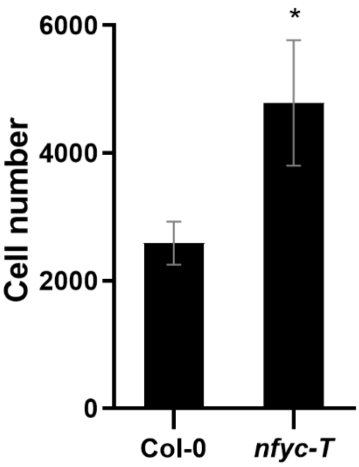

Supplementary Figure S2. Comparison of cell number of first true leaf between *nf-ycT* and Col-0 (two-tailed paired Student's *t*-test; $P \leqslant 0.05$). Data are presented as means ± SD (n = 7).

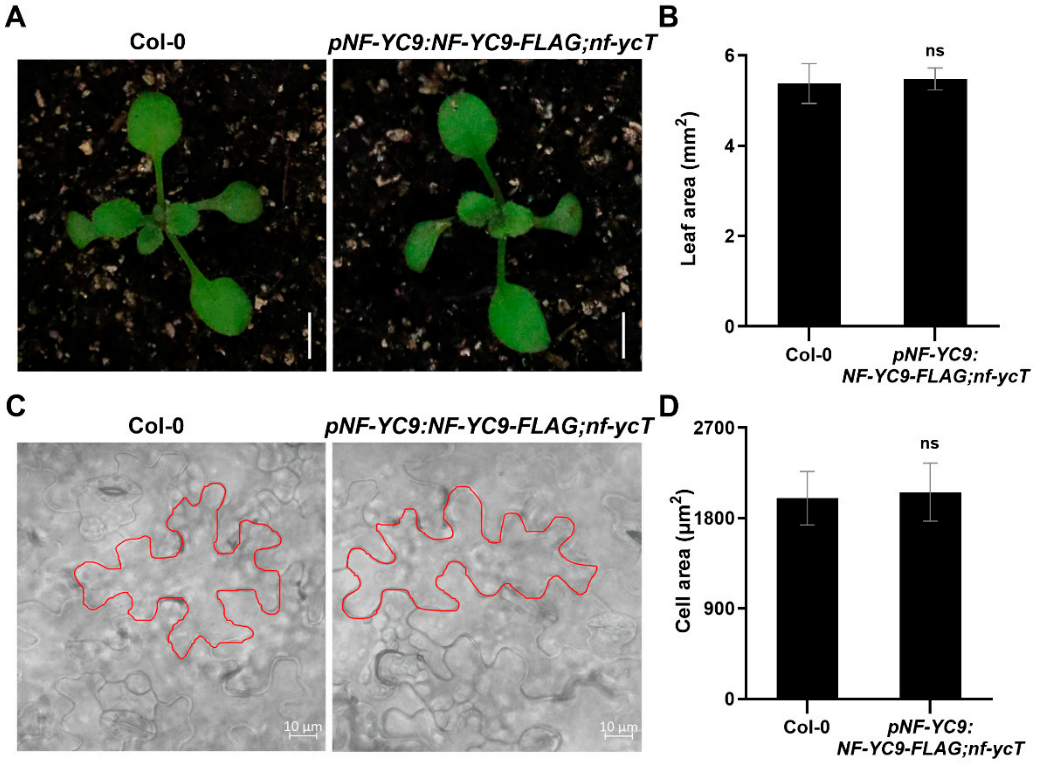

Supplementary Figure S3. Comparison of leaf area and cell area between Col-0 and *pNF-YC9:NF-YC9-FLAG;nf-ycT* line. (A) Morphological observation of whole plants and developing true leaves of Col-0 and *pNF-YC9:NF-YC9-FLAG;nf-ycT* at 14 DAG. Scale bars = 2 mm (B) Statistical analysis of leaf area in *pNF-YC9:NF-YC9-FLAG;nf-ycT* versus Col-0 at 14 DAG (two-tailed paired Student's *t*-test; $P \leqslant 0.05$). Data are presented as means ± SD (n = 7). (C) Microscopic observation of subepidermal cells in the middle region of the first true leaves of Col-0 and *pNF-YC9:NF-YC9-FLAG;nf-ycT* at 14 DAG. Representative intact cells are out-lined by red lines. Scale bars = 10 µm. (D) Comparison of mean cell area in the first true leaves of Col-0 and *pNF-YC9:NF-YC9-FLAG;nf-ycT* at 14 DAG (two-tailed paired Student's *t*-test; $P \leqslant 0.05$). Data are presented as means ± SD (n = 7).
